# Editorial: Organ Fibrosis: Pathogenesis, Biomarkers and Therapeutic Targets

**DOI:** 10.3389/fmed.2021.793507

**Published:** 2021-11-05

**Authors:** Henricus A. M. Mutsaers, Rikke Nørregaard, Peter Olinga

**Affiliations:** ^1^Department of Clinical Medicine, Aarhus University, Aarhus, Denmark; ^2^Department of Pharmaceutical Technology and Biopharmacy, University of Groningen, Groningen, Netherlands

**Keywords:** fibrosis, chronic kidney disease, non-alcoholic fatty liver disease, cirrhosis, pulmonary fibrosis, renal fibrosis, collagen deposition

The prevalence of various chronic and metabolic diseases is on the rise due to the increase in, amongst other factors, lifespan, hypertension, insulin resistance, stress, and obesity. Examples of such diseases include chronic kidney disease and non-alcoholic fatty liver disease (NAFLD), all of which are associated with high levels of morbidity and mortality, and significantly impact health-care budgets. A hallmark of most chronic conditions is the development of fibrosis in the affected organ. Fibrosis is characterized by the excessive production and deposition of extracellular matrix (ECM) proteins, including collagens, mainly by activated (myo)fibroblasts (1). This pathological process has a detrimental impact on organ architecture and function, ultimately necessitating organ transplantation. The fibrotic process is orchestrated by a wide variety of different cells and signaling pathways, which tremendously complicates the identification of relevant biomarkers and druggable therapeutic targets. Consequently, there is still no approved treatment available in clinical practice for most forms of organ fibrosis. Thus, there remains an unmet clinical need. In this Research Topic, experts from all over the world delineate the latest findings pertaining to the pathogenesis of fibrosis as well as arising biomarkers and therapeutic targets.

Interestingly, most contributions to this Research Topic focused on liver fibrosis, which does seem to reflect the recent spike in the number of publications related to this disease indexed in PubMed (Figure 1), whereas in the preceding years (2015–2019) the body of literature concerning liver, renal, cardiac, and lung fibrosis had a fairly similar growth rate.

**Figure 1:**
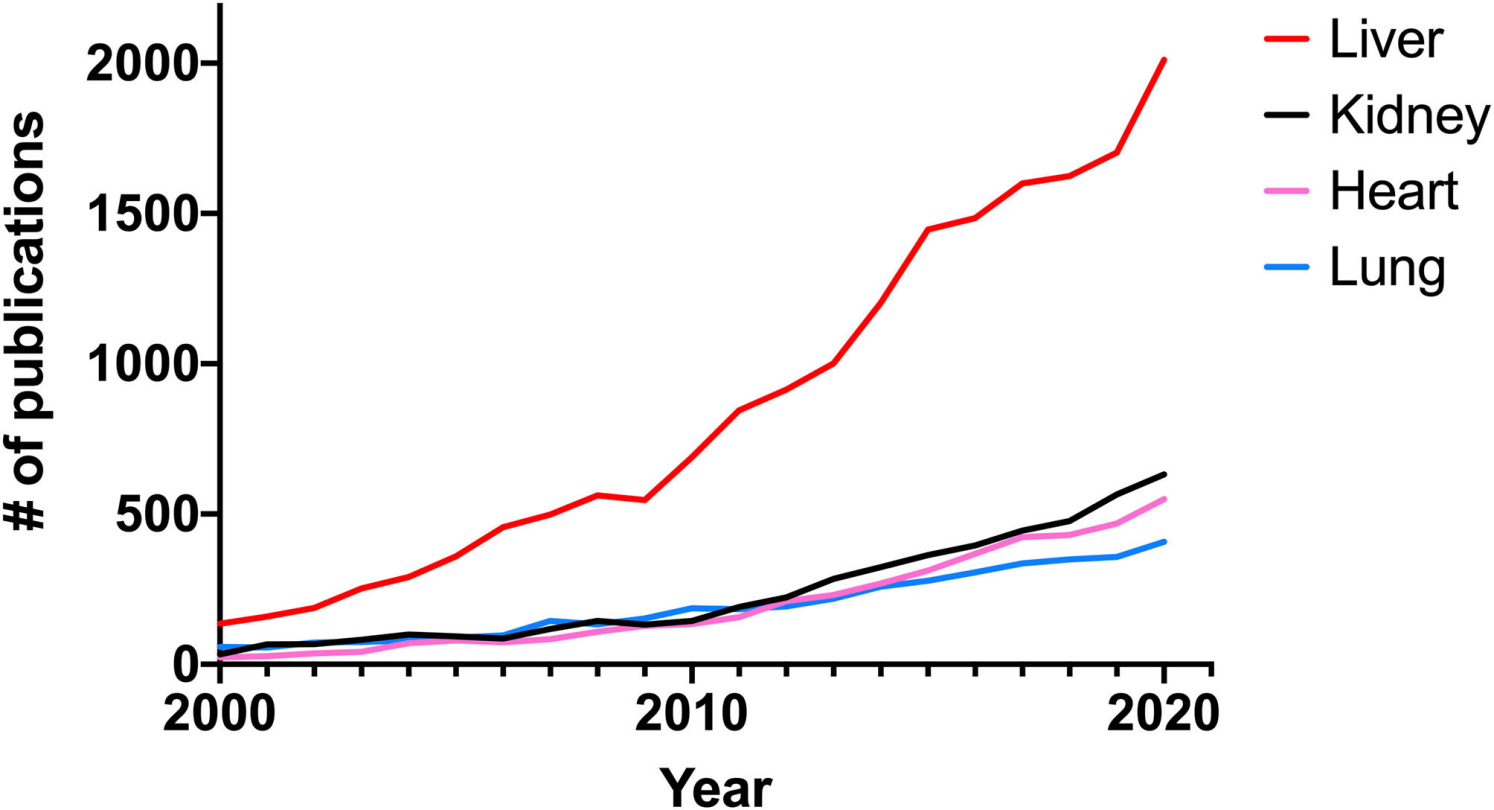
Number of publications related to fibrosis per year indexed in PubMed from 2000 to 2020.

As stated above, the prevalence of NAFLD is on the rise and it currently affects approximately 25% of the world population. Moreover, it is expected that there will be a 178% increase in non-alcoholic steatohepatitis (NASH)-related liver deaths by 2030 (2). Therefore, it is paramount to improve our understanding of NAFLD in order to improve clinical care. The review by Heyens et al., provides a comprehensive overview of the pathological mechanisms underlying the progression from simple steatosis to cirrhosis. In addition, they delineate the current state-of-the-art pertaining to non-invasive tools for diagnosis and monitoring of disease progression and drug responses. Lastly, they report on current and future treatment modalities. This work provides a solid foundation for future work on NAFLD and liver fibrosis. Another aspect of liver fibrosis that is gaining more and more attention from the scientific community is the role of immune cells, especially macrophages, in disease progression. Wan et al. describe in detail the regulatory role of T helper 17 cells, T helper 9 cells, T helper 22 cells, mucosa-associated invariant T cells, regulatory T cells, innate lymphoid cells, and γδ T cells in liver fibrosis. This work highlights the immunological intricacies of the fibrotic process, and reveals that although some work has been done regarding the therapeutic potential of modulating T helper 17 and regulatory T cell responses, more research is clearly needed before we can harness the spectrum of immune cells as therapeutic targets. The work by Gantzel et al., delineates how the scavenger receptors CD163 and mannose receptor (CD206), expressed and secreted by macrophages, can be used as biomarkers to estimate the degree of ongoing inflammation in acute and chronic liver diseases in general and in assessing fibrosis severity in patients with NAFLD as well as chronic hepatitis B and hepatitis C (HCV) infections. These findings were corroborated by the study of Xi et al., who showed that CD163 expression correlated with liver fibrosis progression. Moreover, they demonstrated that CCL2 and TGF-β stimulation increased CD163 expression *in vitro*. However, the combination of CD163 or CD206 with transient elastography (TE) did not appear to improve fibrosis prediction, as compared to TE alone, in two independent cohorts from Italy and Sweden, as shown by Kazankov et al. Thus, the search for highly predictive soluble biomarkers continues. Another marker that might be useful in the clinic is bromodomain-containing protein 4 (BRD4)—a transcriptional co-activator of several pro-fibrotic genes such as collagen 1. Wu et al., reported that BRD4 is up-regulated in liver fibrosis, regardless of etiology, and its expression levels positively correlated with disease severity. However, more research is needed to evaluate whether this marker is superior to currently used tools such as TE. Next to biomarkers, it is also important to identify patient-specific (genetic) risk factors for liver fibrosis, which can contribute to improved and more personalized clinical care. The study by Pineda-Tenor et al. revealed an association between *MTHFR* (methylenetetrahydrofolate reductase; an essential gene for the folate cycle and homocysteine metabolism) rs1801133 polymorphisms and liver fibrosis progression in HCV-infected patients. Their work demonstrated that *MTHFR* rs1801133 C allele carriers presented a diminished risk of liver fibrosis progression as compared to rs1801133 T allele carriers. Even though the study only included a small number of patients, it is a valuable and thought-provoking addition to the current literature regarding the impact of homocysteine homeostasis on NAFLD/NASH and liver fibrosis (3). The final addition to this body of work on liver fibrosis deals with biliary atresia (BA) and matrix metalloproteinase-7 (MMP-7). Nomden et al., provide an extensive overview of the molecular mechanisms of BA and the potential role of MMP-7 herein. As stated by the authors “BA and MMP-7 have an interesting relationship which is yet to be specified,” and their work is an essential part of the foundation for future research into BA pathogenesis.

With regards to pulmonary fibrosis, the review by Ding et al. delineates how neutrophils can contribute to tissue destruction, thereby unveiling potential therapeutic targets related to neutrophil functions, such as the formation of neutrophil extracellular traps, for the treatment of lung fibrosis. The study by Ruigrok et al., demonstrated that silencing heat shock protein 47—a chaperone protein of collagen—did not prevent TGF-β-induced fibrosis in murine precision-cut lung slices. Directly after publication this paper received quite some attention on social media and was applauded for presenting “negative results,” underscoring the fact that the scientific community needs an outlet for this type of work (4).

Models for fibrosis are constantly being improved to enhance translatability. The paper by Puerta Cavanzo et al. describes the use of macromolecular crowders to promote collagen deposition *in vitro* thereby improving the Scar-in-a-Jar system. As such, the model is a great addition to the toolbox for fibrosis research.

All authors contributing to this Research Topic have made a tremendous effort to provide an overview of the current state of the art in the field of fibrosis research. It was a pleasure to work on this Topic and we hope that the articles will provide the readers with a better understanding of fibrosis and showcase the cornucopia of unanswered questions, thereby stimulating new avenues of research.

## Author Contributions

HAMM, RN, and PO conceived and wrote the manuscript. All authors approved the final version of the manuscript and fully agree with its content.

## Conflict of Interest

The authors declare that the research was conducted in the absence of any commercial or financial relationships that could be construed as a potential conflict of interest.

## Publisher's Note

All claims expressed in this article are solely those of the authors and do not necessarily represent those of their affiliated organizations, or those of the publisher, the editors and the reviewers. Any product that may be evaluated in this article, or claim that may be made by its manufacturer, is not guaranteed or endorsed by the publisher.
